# Lowering platelet-count threshold for transfusion in preterm neonates decreases the number of transfusions without increasing severe hemorrhage events

**DOI:** 10.1007/s00431-024-05709-x

**Published:** 2024-08-09

**Authors:** Elodie Billion, Souad Ghattas, Pierre-Henri Jarreau, Roberta Irmesi, Bellaure Ndoudi Likoho, Juliana Patkai, Elodie Zana-Taieb, Heloise Torchin

**Affiliations:** 1 Department of Neonatal Medicine of Port-Royal, Cochin Hospital, FHU PREMA, AP-HP Centre, Université Paris Cité, 75014 Paris, France; 2 Université Paris Cité, Inserm U955, Paris, France; 3 CRESS, Obstetrical Perinatal and Pediatric Epidemiology Research Team, EPOPé, INSERM, INRAE, Université Paris Cité, 75004 Paris, France; 4 Réanimation Néonatale, Hôpital Femme Mère Enfant, 59 Bd Pinel, 69500 Bron, France

**Keywords:** Neonates, Preterm, Platelet transfusion, Threshold, Hemorrhage

## Abstract

Thrombocytopenia is common in preterm neonates and can be associated with hemorrhage. Most platelet transfusions are prophylactic. Previously, higher platelet-count thresholds were recommended for neonates, but this recommendation has been questioned in recent studies. In the PlaNeT2 trial, mortality and serious bleeding were more frequent in neonates with the highest platelet-count threshold than in others. Following this trial, we changed our platelet transfusion practice by lowering the platelet-count threshold for prophylactic transfusion from 50,000 to 25,000/mm^3^. We conducted a before–after retrospective cohort study to quantify the frequency of platelet transfusions and assess the new protocol by analyzing death and serious hemorrhage events. This retrospective monocentric study included neonates born before 37 weeks of gestation with platelet count < 150,000/mm^3^ during the 2 years preceding the new platelet transfusion protocol (high prophylactic transfusion threshold, 50,000/mm^3^) and during the 2 years after the new platelet transfusion protocol (low prophylactic transfusion threshold, 25,000/mm^3^). The primary outcome was the proportion of neonates receiving at least one platelet transfusion in both groups. We also compared the proportion of deaths and severe hemorrhage events. A total of 707 neonates with thrombocytopenia were identified. In the high-threshold group, 99/360 (27.5%) received at least one platelet transfusion as compared with 56/347 (16.1%) in the low-threshold group (*p* < 0.001). The groups did not differ in proportion of deaths or severe hemorrhage events.

*Conclusions*: A reduced platelet-count threshold for transfusion allowed for a significant reduction in the number of platelet transfusions without increasing severe hemorrhage events.

**Table:** 

**What is Known:** • *A recent randomized trial suggested that restrictive platelet-count thresholds for platelet transfusion could be beneficial for preterm neonates.*
**What is New:** • * On lowering the platelet-count threshold for transfusion from 50,000 to 25,000/mm3, the number of transfusions significantly decreased without increasing severe hemorrhage events in a neonatal intensive care unit.*

**Supplementary Information:**

The online version contains supplementary material available at 10.1007/s00431-024-05709-x.

## Introduction

The management of thrombocytopenia (platelet count < 150,000/mm^3^) in neonatal intensive care units (NICUs) involves platelet transfusion and aims at preventing hemorrhage, specifically in very preterm neonates who are at risk of high-grade intraventricular and intra-parenchymal hemorrhage [1–3]. Nearly 10% of the neonates admitted to NICUs receive a platelet transfusion [4, 5]. However, evidence guiding platelet transfusion practices in preterm neonates is sparse and is mainly based on expert opinion. Hence, the platelet-count threshold for transfusion varies considerably among countries, from 20,000 to 100,000/mm^3^ [6–8]. In France, recommendations were updated in 2015 and advocated a threshold of < 30,000/mm^3^ for neonates with non-immune thrombocytopenia and 50,000/mm^3^ for neonates with risk factors [9].

Overall, 90 to 98% of platelet transfusions in NICUs are prophylactic (i.e., prior to any hemorrhage) [10]. However, there is insufficient evidence to show a causal association between platelet count and risk of hemorrhage. Recent studies support lowering the platelet-count threshold for transfusion without increasing the risk of hemorrhage [10–12]. Among these studies, the PlaNeT2 randomized trial compared platelet transfusion thresholds of 25,000 and 50,000/mm^3^ in preterm neonates. Mortality, serious hemorrhage events, and significant neurodevelopmental impairment at 2 years of corrected age were more frequent in neonates with the highest threshold than others [13–15]. Potential complications of platelet transfusion should also be considered [6, 14, 15]. Multiple studies have shown an association between platelet transfusions and neonatal mortality [16–20].

The platelet-count threshold for transfusion was modified in our NICU after the publication of the PlaNeT2 results [13], from a prophylactic platelet-count threshold of 50,000 to one of 25,000/mm^3^ in most cases. We conducted a before–after retrospective cohort study to quantify the frequency of platelet transfusions and assess the new protocol’s tolerance by analyzing death and serious hemorrhage events.

## Methods

### Setting

Our department is a NICU within a type 3 maternity hospital with 5500 births per year. About 1250 neonates per year are admitted to the NICU. All neonates delivered before 34 weeks of gestation (WG) undergo neurosensory investigations, in particular cerebral ultrasonography twice in the first 10 days and at 3 weeks of life. Ultrasonography is performed more often in cases of clinical instability.

### Platelet-count thresholds for transfusion and protocols

The results of PlaNeT2 were presented in a weekly literature review. After that, we updated the platelet transfusion protocol after a discussion with the medical team. Until December 2020, the prophylactic platelet-count threshold for transfusion was 50,000/mm^3^. In cases of surgery or active hemorrhage, the threshold was set at 80,000 to 100,000/mm^3^.

Since December 2020, our local protocol has recommended a platelet-count threshold for transfusion of 25,000/mm^3^. In cases of active hemorrhage or serious hemorrhage in the previous 72 h, lumbar puncture, hemodynamic instability, or gestational age < 26 WG in the first 72 h of life, the threshold is 50,000/mm^3^. Serious hemorrhage was defined as high-grade intraventricular hemorrhage or pulmonary or digestive hemorrhage.

Transfusion modalities are in line with the 2015 French recommendations, that is, apheresis platelet concentrates from a single donor with pediatric processing, deplasmatized in case of alloimmune thrombocytopenia, and treated with amotosalen, respecting the ABO system whenever possible. The volume transfused is 15 to 20 ml/kg or 0.1 to 0.2 × 10^11^ platelets/kg over 60 min. The transfusion is administered by a peripheral venous catheter or the second line of an umbilical venous catheter.

### Study design and population

In this retrospective monocentric study, we included all neonates born at < 37 WG and admitted to the NICU from 1 January 2018 to 31 December 2019 (representing the high-threshold (HT) group) and from 1 January 2021 to 31 December 2022 (representing the low-threshold (LT) group). Neonates were eligible for the study if they presented at least one episode of thrombocytopenia (platelet count < 150,000/mm^3^) during their neonatal stay. Exclusion criteria were false severe thrombocytopenia defined as platelet count < 50,000/mm^3^ and controlled at > 150,000/mm^3^ in the following 12 h without any transfusion.

### Outcomes

The primary outcome was the number of neonates receiving at least one platelet transfusion. Secondary outcomes were the number of transfusions per neonate who received a platelet transfusion and rates of death and severe hemorrhage events within 28 days after a diagnosis of severe thrombocytopenia < 50,000/mm^3^. Severe hemorrhage was defined as rectal hemorrhage, grade 3 or 4 intraventricular hemorrhage according to Papile’s classification (23), pulmonary hemorrhage (fresh bleeding through an endotracheal tube with increased ventilatory requirements), or hemorrhage associated with shock.

### Data collection

The study was declared to the institution’s registry (no. 20220701152308). Parents were informed that their newborn’s data could be used to evaluate medical practices and that they had the right to object. Data were obtained retrospectively from health records and included data on the antenatal period and thrombocytopenia and platelet transfusion. The main neonatal characteristics and outcomes were also recorded. For neonates with severe thrombocytopenia (< 50,000/mm^3^), additional data collected were red blood cell transfusion, complications occurring in the 28 days after the diagnosis of severe thrombocytopenia (digestive hemorrhage, high-grade intraventricular hemorrhage, death) and occurrence of bronchopulmonary dysplasia.

### Statistical analysis

Descriptive statistics were used to summarize the data. Categorical variables are described with number (percentage) and were compared by chi-squared test. Quantitative data are described with mean (SD) and were compared by the Student *t*-test. We compared the perinatal characteristics of infants and biological results concerning platelet counts in both periods, the 2 years preceding the new platelet transfusion protocol (HT threshold) and the 2 years after the new platelet transfusion protocol (LT threshold). We also compared characteristics of neonates receiving at least one platelet transfusion in both periods. We describe the outcomes of infants with severe thrombocytopenia. We compared the characteristics of infants with severe (< 50,000/mm^3^) and moderate (50–150,000/mm^3^) thrombocytopenia. All analyses involved using SAS 9.4 (SAS Institute Inc., Cary, NC, USA) with significance set at *p* < 0.05.

## Results

We identified 707 patients with thrombocytopenia: 360 in the HT group and 347 in the LT group. Baseline characteristics are presented in Table 1. The groups were comparable in characteristics, except for fewer cesarean deliveries and fewer neonates with intra-uterine growth restriction in the HT than in the LT group.

**Table 1:** Characteristics of neonates with high and low platelet-count thresholds

	High threshold (50,000/mm^3^) *n* = 360	Low threshold (25,000/mm^3^) *n* = 347	*p*-value
General characteristics			
Gestational age (WG) (mean, SD)	29.0 (3.8)	29.4 (3.8)	0.21
Gestational age (range)	23–36	23–36	
Gestational age (WG) (*n*/*N*, %)			
< 26	113/360 (31.4)	79/347 (22.8)	0.09
26–28	63/360 (17.5)	70/347 (20.2)	
28–32	89/360 (24.7)	93/347 (26.8)	
≥ 32	95/360 (26.4)	105/347 (30.3)	
Birthweight (g) (mean, SD)	1121 (577)	1166 (577)	0.30
Birthweight (g) (range)	415–3710	460–3225	
Birthweight (g) (*n*/*N*, %)			
< 800	149/358 (41.6)	118/347 (31)	0.16
800–1200	87/358 (24.3)	102/347 (29.5)	
1200–1600	53/358 (14.8)	49/347 (14.2)	
≥ 1600	95/358 (26.4)	77/347 (22.3)	
Intrauterine growth restriction (*n*/*N*, %)	157/358 (43.9)	191/347 (55.0)	< 0.01
Male sex (*n*/*N*, %)	198/359 (55.2)	177/347 (51.0)	0.27
Antenatal characteristics (*n*/*N*, %)			
Multiple pregnancy	82/358 (22.9)	87/346 (25.1)	0.49
Outborn	37/359 (10.3)	48/347 (13.8)	0.15
Cesarean delivery	210/359 (58.5)	230/347 (66.3)	0.03
Antenatal glucocorticoids, ≥ 1 dose	293/342 (85.7)	270/304 (88.8)	0.23
Preeclampsia	98/359 (27.3)	111/347 (32.0)	0.17
Chorioamnionitis	91/359 (25.3)	80/347 (23.1)	0.48

Denominators vary due to missing data

*WG* weeks of gestation

The LT and HT groups had similar mean platelet counts at birth and evolution during the first postnatal days (Table 2). In the HT group, 99/360 (27.5%) patients received at least one platelet transfusion as compared with 56/347 (16.1%) in the LT group (*p* < 0.001), for a relative risk reduction of 41%. The mean number of platelet transfusions per patient was 1.8 (SD 1.6) in the LT group and 2.4 (SD 2.4) in the HT group (*p* < 0.01). This reduction was accompanied by a reduction in volume transfused: mean 27.5 (SD 24.7) ml/kg in the LT group and 35.0 (SD 35.3) ml/kg in the HT group. Additional investigations were conducted for about 40% of the neonates in cases of persistent thrombocytopenia that was not explained by growth retardation or sepsis. The most frequently investigated condition was cytomegalovirus infection, investigated in nearly 40% of all neonates included but positive for only 3.1%.

**Table 2:** Characteristics of thrombocytopenia for neonates with high and low platelet-count thresholds

	High threshold (50,000/mm^3^) *n* = 360	Low threshold (25,000/mm^3^) *n* = 347	*p*-value
Platelet count at birth, /mm^3^ (mean, SD)	179,000 (76,000)	177,000 (74,000)	0.76
Platelet count nadir, /mm^3^ (mean, SD)	83,000 (40,000)	86,000 (42,000)	0.42
Postnatal age at platelet count nadir, days (mean, SD)	7.0 (10.3)	6.8 (9.9)	0.73
Early-onset severe thrombocytopenia (< 3 days) (*n*/*N*, %)	33/360 (9.2)	20/347 (5.8)	0.09
Thrombocytopenia ≤ 50,000/mm^3^ (*n*/*N*, %)	98/359 (27.3)	90/347 (25.9)	0.68
Thrombocytopenia ≤ 25,000/mm^3^ (*n*/*N*, %)	30/359 (8.4)	33/347 (9.5)	0.59
Platelet transfusion (> 1) (*n*/*N*, %)	99/360 (27.5)	56/347 (16.1)	< 0.001

Denominators vary due to missing data

Overall, 98 neonates in the HT group and 90 in the LT group presented severe thrombocytopenia (platelet count < 50,000/mm^3^); for these infants, the mean gestational age was 28.1 (SD 3.3) WG and mean birthweight 957 (SD 489) g. Their characteristics and outcomes, in particular death or digestive or intraventricular hemorrhage, are in Table 3. Fewer infants in the LT than in the HT group had complications in the 28 days after the diagnosis of severe thrombocytopenia (28% vs 42%, *p* = 0.04). Mortality rates were high in both groups: 33.7% in the HT group and 25.6% in the LT group (*p* = 0.22) (i.e., 29.8% of neonates with severe thrombocytopenia). In comparison, the mortality rate was 9.3% for neonates with moderate thrombocytopenia (platelet count 50 to 150,000/mm^3^). No pulmonary hemorrhage event was recorded, and only one neonate from the HT group had a retinal hemorrhage. The occurrence of bronchopulmonary dysplasia, defined as the need for ventilatory support (oxygen or invasive and non-invasive ventilation at 36 WG), was quite high, at 63% among neonates with thrombocytopenia < 50 G/l and comparable in the two groups. However, Table 3 presents data on neonates who had severe thrombocytopenia < 50,000 platelets/mm^3^, which is a marker of overall severity. Among thrombocytopenic children with > 50,000 platelets/mm^3^, the occurrence of bronchopulmonary dysplasia is lower (161/351 or 45.9%).

**Table 3:** Outcomes of neonates with severe thrombocytopenia < 50,000/mm^3^ and high and low platelet-count thresholds whether they received transfusion or not

	All	High threshold (50,000/mm^3^)	Low threshold (25,000/mm^3^)	*p*
	*N* = 188	*N* = 98	*N* = 90	
Complications within 28 days after diagnosis of severe thrombocytopenia (*n*/*N*, %)				
None	116/179 (64.8)	52/90 (57.8)	64/89 (71.9)	0.04
Digestive hemorrhage	2/1479 (1.1)	1/90 (1.1)	1/89 (1.1)	0.99
Grade 3 or 4 intraventricular hemorrhage	4/179 (2.2)	3/90 (3.3)	1/89 (1.1)	0.32
Death	56/188 (29.8)	33/98 (33.7)	23/90 (25.6)	0.22
Red blood cell transfusion (*n*/*N*, %)	121/177 (68.4)	64/88 (72.7)	57/89 (64.0)	0.21
Children alive at 36 weeks postmenstrual age	*N* = 132	*N* = 65	*N* = 67	
Bronchopulmonary dysplasia at 36 weeks postmenstrual age* (*n*/*N*, %)	66/104 (63.5)	32/54 (59.3)	34/50 (68.0)	0.36

Denominators vary due to missing data

^*^Among children alive at 36 weeks

We also compared the baseline characteristics of neonates with severe versus moderate thrombocytopenia (platelet count 50,000 to 150,000/mm^3^); the results are in Table S1, supplementary data. Neonates with severe thrombocytopenia (platelet count < 50,000/mm^3^) were more premature and had a lower birthweight than those with moderate thrombocytopenia.

In most cases, the indication for transfusion was asymptomatic thrombocytopenia under the threshold (Table 4). The two groups were similar in clinical condition at first transfusion, with many neonates having sepsis and requiring invasive ventilation (Table 4). Active hemorrhage occurred in 20.2% of the neonates in the HT group and 35.7% in the LT group. Prior hemodynamic instability requiring inotropic support was more frequent but not significantly in the HT than in the LT group.

**Table 4:** Characteristics of neonates at first platelet transfusion according to high or low platelet-count threshold

	High threshold (50,000/mm^3^) *n* = 99	Low threshold (25,000/mm^3^) *n* = 56	*p*-value
Gestational age, WG (mean, SD) *N*	27.6 (3.1) *99*	27.9 (3.5) *56*	0.53
Postnatal age at first transfusion, days (mean, SD) *N*	6.4 (8.5) *99*	8.5 (11.5) *56*	0.20
Platelet count at first transfusion, /mm^3^ (mean, SD) *N*	41,000 (15,000) *99*	28,000 (14,000) *56*	< 0.001
Postnatal age at last transfusion, days (mean, SD) *N*	11 (12.5) *97*	10 (12) *51*	0.66
Number of platelet transfusions per patient (mean, SD) *N*	2.4 (2.4) *98*	1.8 (1.6) *55*	< 0.01
Volume of transfusion, ml (mean, SD) Volume of transfusion, ml/kg (mean, SD) *N*	41.5 (57.9) 35.0 *99*	28.3 (26.1) 27.5 *55*	0.10 0.13
Indications for transfusion (*n*/*N*, %)			
Asymptomatic thrombocytopenia < threshold	51/99 (51.5)	27/56 (48.2)	0.90
Sepsis	30/99 (30.3)	9/56 (16.1)	
Active hemorrhage	10/99 (10.1)	15/56 (26.8)	
Non-steroidal anti-inflammatory drug	4/99 (4.0)	0/56 (0)	
Unknown	4/99 (4.0)	5/56 (8.0)	
Transfusion with platelet count greater than current threshold (*n*/*N*, %)	14/99 (14.1)	20/56 (35.7)	< 0.01
Clinical condition at first transfusion (*n*/*N*, %)			
Invasive ventilation	59/99 (59.6)	32/55 (57.1)	0.86
Sepsis	54/99 (54.5)	34/56 (60.7)	0.46
Necrotizing enterocolitis	3/98 (3.1)	7/56 (12.5)	0.02
Active hemorrhage	20/99 (20.2)	20/56 (35.7)	0.03
Indication for lumbar puncture	12/98 (12.2)	9/56 (16.1)	0.51
Indication for non-steroidal anti-inflammatory drug	11/98 (11.2)	2/56 (3.6)	0.10
Grade 3 or 4 intraventricular hemorrhage	15/98 (15.3)	11/56 (19.6)	0.49
Disseminated intravascular coagulation	6/99 (6.1)	3/56 (5.4)	0.86
Hemodynamic instability	42/95 (44.2)	16/56 (28.6)	0.06

Denominators vary due to missing data

*IQR* interquartile range, *WG* weeks of gestation

Some neonates (14.4% in the HT group and 20.6% in the LT group) did not meet the platelet-count threshold for transfusion. The mean minimum platelet count was 58,000 (SD 10,000) per cubic millimeter in the HT group and 33,000 (SD 8000) per cubic millimeter in the LT group. Moreover, 12 (3%) neonates in the HT group did not receive a transfusion even though their minimum platelet count was < 50,000/mm^3^. Seven (60%) had a platelet count very close to the threshold, and the platelet count was above the threshold when it was checked a second time. Two neonates had severe neurological damage, and withholding or withdrawing life-sustaining therapies had been decided. Medical records were incomplete for three neonates of the HT group, and we could not record any information about transfusion. In the LT group, one neonate had a minimum platelet count < 25,000/mm^3^ and did not receive transfusion. The minimum platelet count occurred in a sepsis context and was close to the threshold (24,000/mm^3^), with a count of 60,000/mm^3^ 12 h later.

## Discussion

In this monocentric retrospective cohort study, a lower platelet-count threshold for transfusion in preterm neonates allowed for a significant reduction in the number of transfusions without increasing severe hemorrhage events. Rates of platelet transfusions in preterm neonates with thrombocytopenia < 150,000/mm^3^ decreased from 27% in the HT group to 16% in the LT group (i.e., a relative risk reduction of 41%). In both periods, the 2 years preceding the new platelet transfusion protocol (HT threshold) and the 2 years after the new platelet transfusion protocol (LT threshold), severe thrombocytopenia < 50,000/mm^3^ was associated with high mortality.

However, the change in platelet-count threshold for transfusion was not associated with increased rates of death or severe hemorrhage. This finding in our real-world study agrees with the PlaNeT2 trial results. In this trial, which included infants with platelet count < 100,000/mm^3^, 90% of the neonates in the HT group and 53% in the LT group received at least one platelet transfusion (i.e., a relative risk reduction of 41% as well). Moreover, morbi-mortality was less frequent in the LT than in the HT group [13]. A recent study, also inspired by the PlaNeT2 trial, did not find a reduction in the number of platelet transfusions after a change in platelet transfusion guidelines [22]. However, the study maintained a threshold of 50,000/mm^3^ for neonates at risk of intra-ventricular hemorrhage, which concerns many neonates in a NICU. In contrast, in another study, restrictive local transfusion thresholds in a NICU decreased the proportion of platelet-transfused neonates by 46% [23].

Although the protocol with a lower threshold was generally well applied, 36% of the transfusions were performed out of protocol, more in the LT than in the HT group. The new protocol was implemented in December 2020, and our study began in January 2021 so that medical staff could get used to it. To raise awareness among the medical team about the change in the platelet transfusion protocol, we requested that each indication for platelet transfusion be documented in the patient’s record. This gave us the opportunity to reiterate the importance of restrictive transfusion practices. However, staff may have been concerned that the platelet count would quickly fall below the threshold, or they may have wished to avoid repeated blood samples, therefore performing more transfusions. This observation is consistent with the previous study of transfusions [23], finding an adherence to the new protocol of about 70%. To reduce non-indicated transfusions, the Boston Children’s Hospital NICU set up a key driver diagram to improve the staff’s educational effort and encourage staff buy-in [24]. This program included strengthening the education of providers and nursing staff about the need to use restrictive platelet transfusion thresholds, reminding them daily of the protocol change and discussing situations that required clarification. In our case, reviewing evidence about the potential harm of the use of the liberal transfusion threshold and the poor evidence of an association between low platelet count and intra-ventricular hemorrhage would improve adherence to the new protocol.

Recent studies question the association between thrombocytopenia and hemorrhage in neonates, pointing to the limited effectiveness of platelet transfusions in reducing the risk of hemorrhage and even suggesting that platelet transfusions may be harmful [14, 16–21, 26]. One hypothesis is the potential induction of an inflammatory process when neonates are transfused with adult platelets [25]. Platelets may also promote excessive angiogenic signals during a vulnerable period of brain development [27]. Transfusion-related harm may involve hemodynamic parameters, and fast volume expansion after platelet transfusion administration may disrupt blood flow in the brain and increase the risk of hemorrhage [28]. As a result, platelet count may not be the only factor to consider when prescribing transfusion, and some authors suggest that platelet mass and time of occlusion may better predict the risk of hemorrhage [29, 30].

Our study has some limitations. First, it was a single-center retrospective study, which limits the generalization of our results. We also had missing data, for example, the rate of missing data for bronchopulmonary dysplasia is significant (24.5% in the overall population, 21.2% in the population with thrombocytopenia < 50,000/mm^3^) due to transfers before 36 WG to other hospitals. In these cases, the precise respiratory status at 36 WG was not available. Additionally, the occurrence of bronchopulmonary dysplasia is likely overestimated because transferred neonates generally have a more favorable respiratory outcome than non-transferred neonates. We did not record other aspects of clinical practice that could have changed between the two periods. However, if changes of clinical practice occurred, they probably had a low impact on the risk of thrombocytopenia or major bleeding. Although our study is one of the largest real-world studies of neonates with thrombocytopenia, our sample size might still be too small to draw conclusions about rare side effects.

Our study highlights that lowering the platelet-count threshold for transfusion may result in transfusion savings without increasing complications. This is also the conclusion of a recent meta-analysis of platelet transfusion that recommended the use of restrictive platelet-count thresholds for transfusion in neonates [28]. Platelet transfusion modalities such as volume and duration are still being actively studied and may evolve in the coming years [31].

## Conclusion

The findings of this retrospective study suggest that lowering the platelet-count threshold for transfusion in neonates born < 37 WG is associated with fewer transfusions and seems safe. These real-life results are in line with the current trend to reduce the number of platelet transfusions.

## Supplementary Information

Below is the link to the electronic supplementary material.

Supplementary file1 (DOCX 25 KB)

## Data Availability

No datasets were generated or analysed during the current study.

## Abbreviations

CMV: Cytomegalovirus
IQR: Interquartile range
HT: High threshold
LT: Low threshold
NICU: Neonatal intensive care unit
PCR: Polymerase chain reaction
WG: Weeks of gestation
