# Supplementary material for: Lowering platelet-count threshold for transfusion in preterm neonates decreases the number of transfusions without increasing severe hemorrhage events
Source: Eur J Pediatr. 2024 Aug 9;183(10):4417–24. doi: 10.1007/s00431-024-05709-x (PMC11413073; doi:10.1007/s00431-024-05709-x)
Supplement: Supplementary file 1 — Supplementary file1 (DOCX 25 KB) [file 431_2024_5709_MOESM1_ESM.docx]

**Table S1: Comparison of neonates with moderate and severe thrombocytopenia**

|  | Moderate thrombocytopenia  50,000-150,000 platelets/mm3 | Severe thrombocytopenia <50,000 platelets/mm3 | p-value |
| --- | --- | --- | --- |
|  | n=518 | n=188 |  |
| Gestational age, WG (mean, SD)  *N* | 29.6 (3.8)  517 | 28.1 (3.3)  188 | <0.001 |
| Birthweight, g (mean, SD)  *N* | 1211 (591)  *517* | 957 (489)  *186* | <0.001 |
| Male sex (n/N, %) | 272/517 (52.6) | 102/188 (54.3) | 0.70 |
| Multiple pregnancy (n/N, %) | 131/515 (25.4) | 38/188 (20.2) | 0.15 |
| Chorioamnionitis (n/N, %) | 131/517 (25.3) | 40/188 (21.3) | 0.27 |
| Pre-eclampsia (n/N, %) | 145/517 (28.0) | 63/188 (33.5) | 0.16 |
| Twin-to-twin transfusion syndrome (n/N, %) | 9/516 (1.7) | 5/186 (2.7) | 0.43 |
| Intrauterine growth retardation (n/N, %) | 247/517 (47.8) | 100/187 (53.5) | 0.18 |
| Maternal thrombocytopenia (n/N, %) | 11/81 (13.6) | 6/49 (12.2) | 0.83 |
| Course of glucocorticoids ≥1 dose (n/N, %) | 398/473 (84.1) | 164/172 (95.3) | <0.001 |
| Cesarean delivery (n/N, %) | 315/517 (60.9) | 124/188 (66.0) | 0.22 |
| Outborn (n/N, %) | 63/517 (12.2) | 22/188 (11.7) | 0.86 |
| Platelet count at birth, /mm3 (mean, SD)  *N* | 181,000 (66,000)  *485* | 171,000 (95,200)  *186* | 0.14 |
| Platelet count nadir, /mm3 (mean, SD)  *N* | 103,000 (28,800)  *518* | 31,000 (13,700)  *188* | <0.001 |
| Postnatal age at platelet count nadir, days (mean, SD)  *N* | 6.3 (9.9)  *514* | 8.7 (10.5)  *188* | 0.02 |
| Positive CMV PCR (n/N, %) | 4/170 (2.4%) | 4/88 (4.5%) | 0.33 |

CMV = cytomegalovirus; PCR: polymerase chain reaction, WG= weeks of gestation

Denominators vary due to missing data
